# Development of PDA Nanoparticles for H9N2 Avian Influenza BPP-V/BP-IV Epitope Peptide Vaccines: Immunogenicity and Delivery Efficiency Improvement

**DOI:** 10.3389/fimmu.2021.693972

**Published:** 2021-07-27

**Authors:** Yongqing Liu, Xiaoli Wang, Jiangfei Zhou, Shuaibing Shi, Tengfei Shen, Liangliang Chen, Min Zhang, Chengshui Liao, Chen Wang

**Affiliations:** ^1^The Key Lab of Veterinary Biological Products, Henan University of Science and Technology, Luoyang, China; ^2^School of Basic Medical Sciences, Henan University of Science and Technology, Luoyang, China

**Keywords:** polydopamine, nanoconjugates, nano BPP-V epitope vaccine, nano BP-IV epitope vaccine, H9N2 avian influenza virus

## Abstract

The protection of current influenza vaccines is limited due to the viral antigenic shifts and antigenic drifts. The universal influenza vaccine is a new hotspot in vaccine research that aims to overcome these problems. Polydopamine (PDA), a versatile biomaterial, has the advantages of an excellent biocompatibility, controllable particle size, and distinctive drug loading approach in drug delivery systems. To enhance the immunogenicities and delivery efficiencies of H9N2 avian influenza virus (AIV) epitope peptide vaccines, PDA nanoparticles conjugated with the BPP-V and BP-IV epitope peptides were used to prepare the nano BPP-V and BP-IV epitope peptide vaccines, respectively. The characteristics of the newly developed epitope peptide vaccines were then evaluated, revealing particle sizes ranging from approximately 240 to 290 nm (PDI<0.3), indicating that the synthesized nanoparticles were stable. Simultaneously, the immunoprotective effects of nano BPP-V and BP-IV epitope peptide vaccines were assessed. The nano BPP-V and BP-IV epitope vaccines, especially nano BP-IV epitope vaccine, quickly induced anti-hemagglutinin (HA) antibody production and a sustained immune response, significantly promoted humoral and cellular immune responses, reduced viral lung damage and provided effective protection against AIV viral infection. Together, these results reveal that PDA, as a delivery carrier, can improve the immunogenicities and delivery efficiencies of H9N2 AIV nano epitope vaccines, thereby providing a theoretical basis for the design and development of PDA as a carrier of new universal influenza vaccines.

## Introduction

H9N2 avian influenza virus (AIV), is a low-pathogenicity virus that has attracted substantial attention due to its wide host range (1), high level of genetic diversity due to reassortment (2) and avian-to-human transmission (3), is a serious threat to the poultry industry and human health (4). Vaccination is the most effective measure to prevent influenza virus infections (5). However, antigenic drifts and shifts allow viruses to evade the immune systems to their hosts, resulting in mismatches and low vaccine effectiveness (6), and a universal influenza vaccine is needed. A previous study has shown that conserved epitopes in different influenza virus strains are very promising as vaccine immunogens (7). The immune responses induced by conserved antigens are usually weak, and adjuvants are needed to enhance their potency. In addition, delivery of protein antigens is also challenging due to their fast degradation and diffusion (8).

In recent years, the use of nanoparticles as efficient drug carriers has attracted substantial attention. M2 is a candidate immunogen that is fused to the hepatitis B virus core protein (HBc) and assembled into viral-like particles (VLPs, H2HBc particles). M2 epitopes are exposed on the H2HBc particle surfaces, thereby enabling detection by the immune system and stimulating broad-spectrum, long-lasting protection against influenza A infections (9). A ferritin nanoparticle vaccine that prepared by the coupling of ferritin with preS1 domain of the large HBV surface protein was shown to deliver preS1 to specific myeloid cells and induce a substantial and persistent anti-preS1 response, thereby resulting in efficient viral clearance in a chronic HBV mouse model (10).

Polydopamine (PDA) microsphere, black organic biopolymers, are synthesized by the self-polymerization of dopamine hydrochloride under oxidative and alkaline conditions. The size of the generated PDA nanoparticles can be controlled by the concentration of the free dopamine compound and the rate of hydrogen abstraction (11). Particle size and surface modifications are critical to the design of polymeric particles for therapeutics, as size plays a key role in the overall uptake, distribution, metabolism and elimination of particles. Moreover, increasing the surface area-to-volume ratio can improve nanoparticle uptake (12). In addition, the use of PDA-modified nanoparticles as drug carriers is desirable because of their excellent biocompatibility, mild synthesis requirements, distinctive drug loading approach, and reactive oxygen species (ROS) scavenging ability (13). At present, PDA nanoparticles are widely utilized in tumor-targeted drug delivery system (14). Nanodelivery systems can reduce the exposure of drugs to nontarget sites through targeted delivery, thereby reducing the toxic side effects and multidrug resistance and improving bioavailability (15, 16). However, the application of PDA nanoparticles in influenza vaccines has rarely been reported.

Our researches have shown that BPP-V and BP-IV are used as immune adjuvants in combination with the commercial H9N2 AIV inactivated vaccine to enhance humoral and cellular immune responses (17, 18). Then, BPP-V and BP-IV were bound to the H9N2 AIV epitope peptide to form BPP-V epitope peptide and BP-IV epitope peptide and studied as molecular immune adjuvants. Considering the immunogenicity of the H9N2 AIV epitope vaccine and the advantages of PDA, PDA nanoparticles were herein conjugated with BPP-V and BP-IV epitope peptides to prepare nano BPP-V and BP-IV epitope peptide vaccines with enhanced the immunogenicities and delivery efficiencies. Mouse immune challenge protection experiments were performed to evaluate the immune effects of the nano BPP-V and BP-IV epitope peptide vaccines.

## Materials and Methods

### Peptides, Vaccine, Viruses, Animals, and Reagents

The epitope peptide was obtained by predicting the T and B cell epitopes of the H9N2 AIV haemagglutinin (HA) protein using immunoinformatics methods. The epitope peptide, BPP-V epitope peptide, and BP-IV epitope peptide were synthesized in the solid phase and had purities ≥ 95%. The inactivated H9N2 AIV vaccine (SDS696 strains, 10^7^ median tissue culture infection dose (TCID_50_)/0.1 mL) was purchased from Qian Yuan Hao Biological Co., Ltd. (China). AIV [A/chicken/Jiangsu/JS-1/2002(H9N2)] was maintained in our laboratory (19). Female BALB/c mice (4-6 weeks, 20 ± 2 g) were purchased from Henan Province Experimental Animal Center.

Dopamine hydrochloride was purchased from Shanghai Darui Finechemical Co., Ltd (China). Mouse lymphocyte separation liquid, concanavalin A (ConA), lipopolysaccharide (LPS), thiazolyl blue tetrazolium bromide (MTT), dimethyl sulfoxide (DMSO), and bovine serum albumin (BSA) were obtained from Zhengzhou Jiushi Biotechnology Co., Ltd. (China). Foetal bovine serum (FBS) was purchased from Sijiqing Biotech (China). Taq polymerase was purchased from Takara Biomedical Technology Co., Ltd. (China). The horseradish peroxidase (HRP)-labelled goat anti-mouse IgG antibody and 3,3’,5,5”-tetramethylbenzidine (TMB) were purchased from Boshide Corporation (China). The HRP-conjugated goat anti-mouse IgG1, HRP-conjugated goat anti-mouse IgG2a, fluorescein isothiocyanate (FITC)-labelled anti-mouse CD3, phycoerythrin (PE)-labelled anti-mouse CD4, and PE-labelled anti-mouse CD8 antibodies were obtained from Caltag Corporation (China). Dulbecco’s modified Eagle’s medium (DMEM) and Roswell Park Memorial Institute (RPMI) 1640 medium were obtained from Gibco Corporation (USA). Cytokine detection kits were obtained from eBioscience (USA). The cytotoxicity assay kit was purchased from Promega (USA).

### Preparation of the Nano BPP-V/BP-IV Epitope Peptide Vaccines

**Figure 1:**
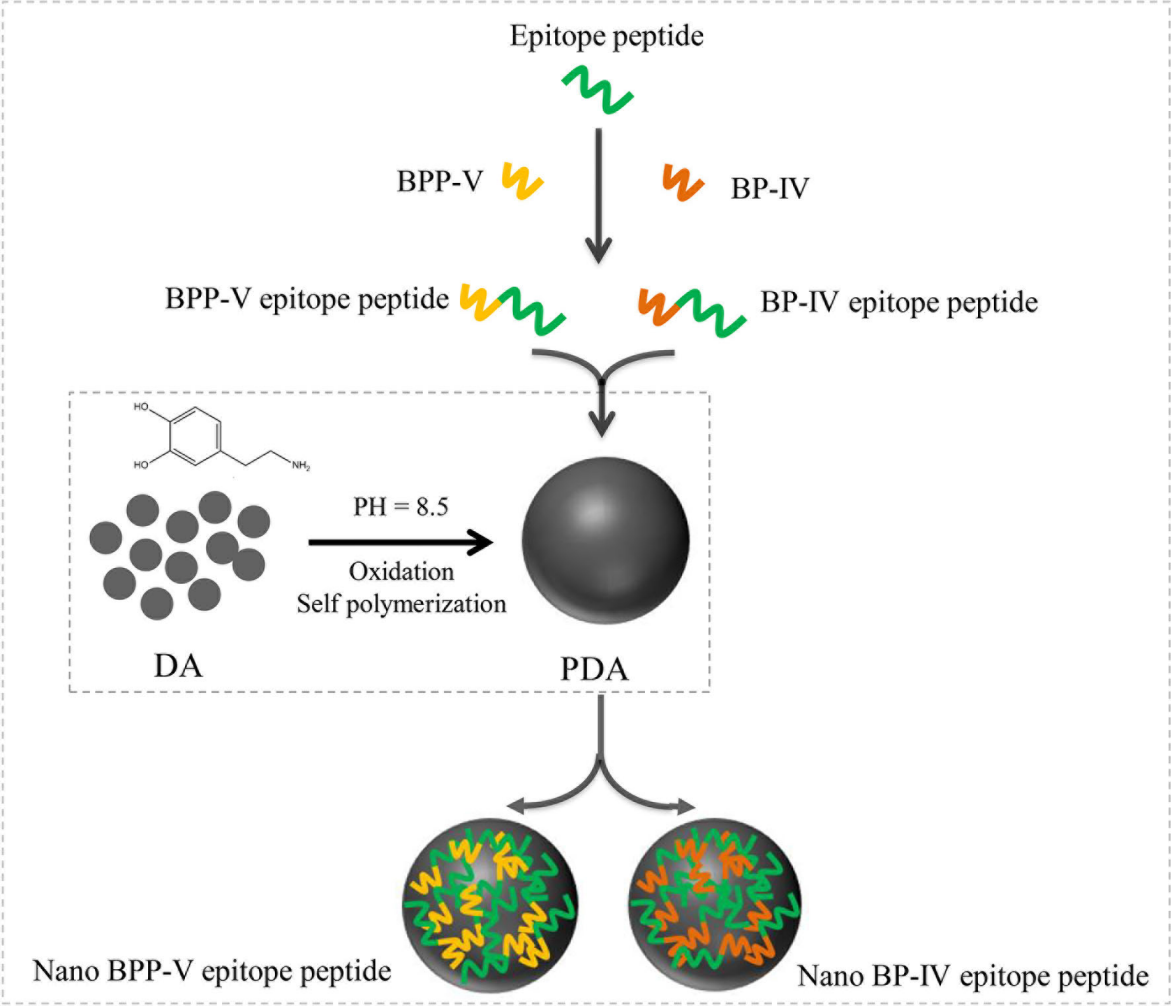
Process diagram of nano BPP-V and BP-IV epitope peptide construction. The epitope peptide was selected by immunoinformatics analysis. As immune adjuvants, BPP-V and BP-IV were conjugated with the epitope peptide to form the BPP-V and BP-IV epitope peptides. Then, the BPP-V and BP-IV epitope peptides were coupled with polydopamine (PDAs microspheres) to form the nano BPP-V and BP-IV epitope peptides vaccines. PDA, a new versatile biomaterial, was synthesized by the oxidation and self-polymerization of dopamine hydrochloride under alkaline conditions.

PDA nanoparticles were prepared as previously described (11, 20). Dopamine hydrochloride (30 mg) and 120 μL NaOH (1 mol/L) were dissolved in deionized water at a total volume of 15 mL and stirred at 50°C. The sample was retrieved by centrifugation (18000 rpm) and washed with deionized water three times. The PDA nanoparticles were obtained by low-speed centrifugation (4000 rpm) to remove all large-size materials. Then, 10 mg of PDA nanoparticles obtained above were dispersed in 10 mL of deionized water. Under vigorous stirring, 1 mg of epitope peptide, BPP-V epitope peptide, BP-IV epitope peptide (1 mg/mL) were added respectively, stirred for 5 h and then centrifuged (18000 rpm). The nanoparticles were retrieved and washed with deionized water three times, and all large-size material were removed by low-speed centrifugation (4000 rpm). Finally, 1 mL of the solution was dried at 80°C and weighed. The free peptides were separated from the nanoparticles by ultracentrifugation and quantified at 480 nm by a UV spectrophotometer (Shimadzu, Japan). The peptide encapsulation efficiency and peptide loading capacity in the nanoparticles were calculated using the following equations: Peptide encapsulation efficiency(wt%)=(mass of the peptide in nanoparticles)/(mass of the peptide in feed)×100%. Peptide loading content(wt%) = (mass of the peptide in nanoparticles)/(the total mass of the nanoparticles) ×100%. The process diagram is shown in **Figure 1**. The nano epitope peptide vaccine, nano BPP-V epitope peptide vaccine, and nano BP-IV epitope peptide vaccines were prepared and stored at 4°C.

### Nanoparticles Characterization

PDA and the nano epitope peptide vaccine, nano BPP-V epitope peptide vaccine, and nano BP-IV epitope peptide vaccine were dissolved in distilled water by ultrasonication, and their morphologies, UV-vis spectra, size distributions, and zeta potentials were determined (21). The morphological characteristics were examined on a Philips CM20 transmission electron microscope (Netherlands), and the UV-vis spectra were recorded by a Perkin-Elmer Lambda 750 instrument (USA). Particle size distribution and zeta potential were measured by using a Malvern ZetaSizer Nano-ZS Zen3600 instrument (UK).

### Animal Immunization

**Figure 2:**
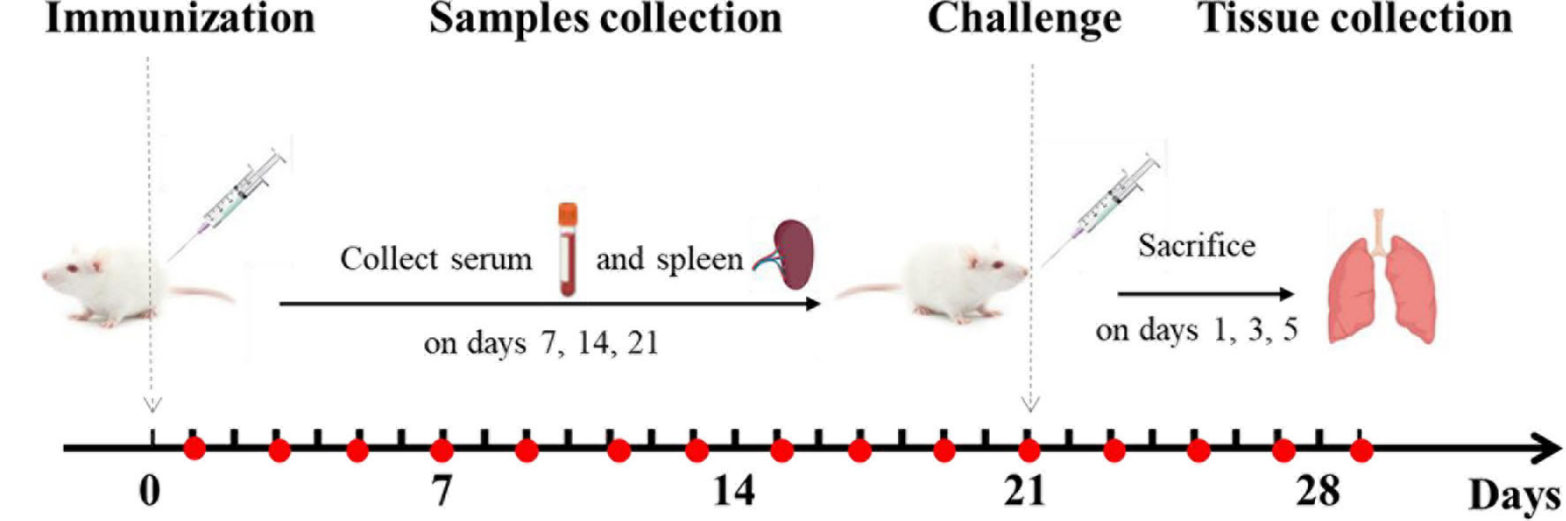
Animal immunization and challenge. Mice were immunized on day 0 and challenged on day 21. Mouse serum was collected for the detection of antibody levels on days 1, 3, 5, 7, 9, 11, 13, 15, 17, 19, 21, 23, 25, 27, and 29 after immunization. The levels of anti-HA (IgG), IgG1, IgG2a, IL-4, and IFN-γ were detected on days 7, 14, and 21 after immunization. Mouse spleens were collected to assess lymphocyte proliferation activity, CTL responses, and T cell subsets on days 7, 14, and 21 after immunization. The lungs of mice were collected to detect the lung viral loads and pathological changes on days 1, 3, and 5 after the challenge.

Mice were randomly divided into the following 9 immunization groups with 40 mice in each group (**Table 1**): PBS (0.2 mL), PDA (0.2 mL), epitope peptide (1 mg/mL, 0.2 mL), epitope peptide vaccine (1 mg/mL, 0.2 mL), nano epitope peptide vaccine (1 mg/mL, 0.2 mL), nano BPP-V epitope peptide vaccine (1 mg/mL, 0.2 mL), nano BP-IV epitope peptide vaccine (1 mg/mL, 0.2 mL), AIV vaccine (0.2 mL), and nonimmune/nonchallenged blank control. The immunizations were performed on day 0 by subcutaneous multisite injection into the back, and the challenge was performed on day 21 by intranasal inoculation (**Figure 2**).

**Table 1:** Animal immunization protocols.

Group	Immunogen (vaccination on day 0)	Dose (Per mouse)
1	PBS	0.2 mL
2	PDA	0.2 mL
3	Epitope peptide (1mg/mL)	0.2 mL
4	Epitope peptide vaccine (1mg/mL)	0.2 mL
5	Nano epitope peptide vaccine (1mg/mL)	0.2 mL
6	Nano BPP-V epitope peptide vaccine (1mg/mL)	0.2 mL
7	Nano BP-IV epitope peptide vaccine (1mg/mL)	0.2 mL
8	AIV vaccine	0.2 mL
9	/	/

Epitope peptide vaccine was prepared with a mixture of epitope peptide and white oil adjuvant./represent the blank control group with no immunity and no challenge. The PDA group was used as a control group. 1mg/mL refers to the concentrations of the peptides. After the calculation of peptide encapsulation efficiency and peptide loading capacity, ultimately, the concentrations of Nano epitope peptide vaccine, Nano BPP-V epitope peptide vaccine, and Nano BP-IV epitope peptide vaccine were also 1mg/mL.

### Detection of Specific Antibodies

Mouse serum samples were collected through tail vein from each group on days 1, 3, 5, 7, 9, 11, 13, 15, 17, 19, 21, 23, 25, 27, and 29 after immunization, and the anti-HA (IgG) antibody were determined to evaluate whether the mice consistently produce antibody. On days 7, 14, and 21 after immunization, mice serum samples were also collected through tail vein, and anti-HA antibody (IgG), IgG1, and IgG2a levels were determined by ELISA as previously described (22). Briefly, 96-well plates were coated overnight at 4°C with recombinant H9N2 HA protein (100 μL per well, 10 μg/mL) and blocked with 1% BSA for 1 h at 37°C. Then, 100 μL of serially diluted serum was added to each well and incubated for 1 h at 37°C. The plates were washed 3 times, incubated with 100 μL of HRP-conjugated goat anti-mouse IgG, IgG1, and IgG2a for 1 h, and washed again. Next, 100 μL of TMB was added for 20 min at 37°C, and 100 μL of 1% SDS was then added to stop the reaction. The optical densities (ODs) were read at 450 nm, and titres at the half-maximal OD were determined by linear interpolation. The experiments were repeated in triplicate.

### Lymphocyte Proliferation Assay

The spleens of mice from each group were collected on days 7, 14, and 21 after immunization and the splenic lymphocyte proliferation assay was performed using the MTT method (17). Briefly, splenic lymphocyte suspensions (2×10^6^ cells/mL) were dispersed and incubated in 96-well plates (100 μL per well), and 50 μL of ConA (40 μg/mL) or LPS (1 μg/mL) was added to each well and incubated at 37°C and 5% CO_2_ for 48 h. Then, 20 μL of MTT (5 mg/mL) was added to each well for 4 h, followed by the addition of 100 μL of DMSO while shaking for 10 min. The OD was read at 570 nm, and the experiments were repeated in triplicate.

### Cytokine Assays

Mouse serum samples were collected from each group on days 7, 14, and 21 after immunization. The secreted levels of IL-4 and IFN-γ in immunized mouse serum were measured by ELISA kits (eBioscience, USA) according to the manufacturer’s instructions.

### Cytotoxic T Lymphocyte Assay

Spleens were harvested from the mice in each group on days 7, 14, and 21 after immunization, and the CTL activity of splenic lymphocytes against NIH3T3 cells was determined by CTL assays (19). Briefly, a splenic lymphocyte suspension (2×10^6^ cells/mL) was prepared as previously described and collected as effector cells (23). NIH 3T3 cells (24) infected with H9N2 AIV (MOI of 20 PFU/cell) for 24 h were used as the target cells. The effector cells were washed 3 times and plated in 96-well plates in RPMI 1640 medium containing 10% FBS. The target cells were added to each well, and the effector-target cell ratios were adjusted to 30:1 and 10:1. The percentage of lysed target cells was measured according to the manufacturer’s instructions (Promega, USA).

### Detection of T Cell Subsets

Spleens were harvested from the mice in each group on day 21 after immunization, and T cell subtype expression (CD3^+^, CD3^+^CD4^+^, and CD3^+^CD8^+^) was detected by flow cytometry as previously described (25). Briefly, spleen lymphocyte suspensions (2×10^6^ cells/mL) were incubated with FITC-labelled CD3^+^ (5 μL) to allow complex formation and then incubated with PE-labelled CD4^+^ and CD8^+^ (5 μL) at 4°C for 1 h. Fluorescence changes were monitored on a Becton Dickinson FACScan flow cytometer to analyze cell expression.

### Animals

Mice were challenged with H9N2 AIV (10^7^ TCID_50_/0.1 mL, 100 μL) by intranasal inoculation on day 21 after immunization. Five mice in each group were randomly sacrificed, and their lung tissues were collected on days 1, 3 and 5 after the challenge. The viral genome copy numbers in lung tissues were determined by real-time PCR (26). The PCR primers were designed based on the HA gene sequence of H9N2 AIV in GenBank (accession no. AY364228) and synthesized by Huada Gene Technology Co., Ltd. Amplification was performed using SYBR green as previously described. The standard curve for real-time PCR quantification was constructed using the HA gene in the pET32a-HA vector. Viral titres in lung tissues were measured by the TCID_50_ assay as previously described (27). Briefly, mice lungs were homogenized in virus growth medium (VGM) (10% wt/vol), tenfold serial dilution of samples were added in MDCK cells and incubated at 37°C for 2 h. Then, fresh VGM was added to the cells and incubated at 37°C for another 48 h. Combined with hemagglutination inhibition test, the log_10_ TCID_50_ per milliliter of lung tissue was calculated as the virus titre by the Reed-Muench method.

In addition, lung tissues were collected on days 1, 3 and 5 after the challenge and fixed in buffered formaldehyde solution for histopathological assessment by hematoxylin and eosin (H&E) staining. The histopathological changes in lung sections were also evaluated based on a standard scoring protocol as previously described (28). The scoring standard was as follows: 0, no pathology; 1, local pulmonary congestion and inflammatory cell infiltration; 2, pulmonary congestion and inflammatory cell infiltration affecting 10% of the section; 3, pulmonary congestion and inflammatory cell infiltration affecting 20% of the section; 4, pulmonary congestion and inflammatory cell infiltration affecting 20-50% of the section; and 5, pulmonary congestion and inflammatory cell infiltration affecting > 50% of the section.

### Statistical Analysis

Experimental data are presented as the mean ± SD. Differences between groups were analyzed using the one-way analysis of variance (ANOVA) followed by LSD’s test for multiple comparisons (SPSS 20.0, IBM Corp., Armonk, NY), and statistical significance was considered at *P* < 0.05 or *P* < 0.01. The sections were observed using Panoramic Viewer software.

## Results

### The Morphological Characteristics, UV-vis Spectra, Particle Size and Zeta Potential

**Figure 3:**
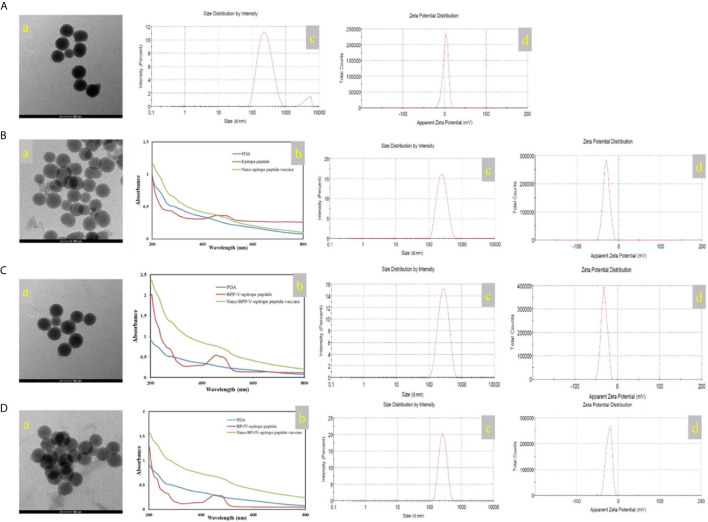
Characterization of the nano vaccines. **(A)** PDA. **(B)** Nano epitope peptide vaccine. **(C)** Nano BPP-V epitope peptide vaccine. **(D)** Nano BP-IV epitope peptide vaccine. a, TEM images; b, UV-vis spectra; c, particle size distribution; d, zeta potential.

In this study, the mass of the epitope peptide, BPP-V epitope peptide, and BP-IV epitope peptide in nanoparticles were 0.65 mg, 0.45 mg, and 0.5 mg, respectively. The mass of all three peptides in feed were 1.0 mg, and the total mass of the nanoparticles were 10 mg. So that epitope peptide encapsulation efficiency is 65% and epitope peptide loading content is 6.5%. The BPP-V epitope peptide encapsulation efficiency is 45% and BPP-V epitope peptide loading content is 4.5%. The BP-IV epitope peptide encapsulation efficiency is 50% and BP-IV epitope peptide loading content is 5%. PDA was formed into a black organic biopolymer by dopamine oxidation and self-polymerization under basic oxygen conditions as determined by transmission electron microscopy (TEM). PDA nanoparticles were bound to the H9N2 AIV epitope peptide coupled with BPP-V or BP-IV to form the nano BPP-V and BP-IV epitope vaccines, which also exhibited spherical organic polymers (**Figures 3A–D**). The UV-vis absorption spectra of the epitope peptides exhibited a peak at approximately 500 nm, while PDA by itself did not, but the nano epitope peptide vaccines also exhibited a peak at approximately 500 nm, indicating that PDA and the epitope peptides were combined successfully. The sizes and zeta potentials of the nano epitope vaccines were also analyzed. The PDA nanoparticle had an average diameter of approximately 239 nm (PDI=0.256) with a membrane zeta potential of 2.12 mV. The average particle size of the nano epitope peptide vaccine was 242.9 nm (PDI=0.159), with a narrow particle size distribution and a zeta potential of -29.4 mV. The average particle size of the nano BPP-V and BP-IV epitope vaccines was approximately 270-290 nm (PDI<0.3), and the zeta potential was approximately -20 ~ -30 mV, indicating that the nano BPP-V and BP-IV epitope vaccines were synthesized successfully and the synthesized PDA nanoparticles were stable (**Figures 3C, D**).

### Nano BPP-V and BP-IV Epitope Peptide Vaccines Can Rapidly Stimulate Antibody Production

**Figure 4:**
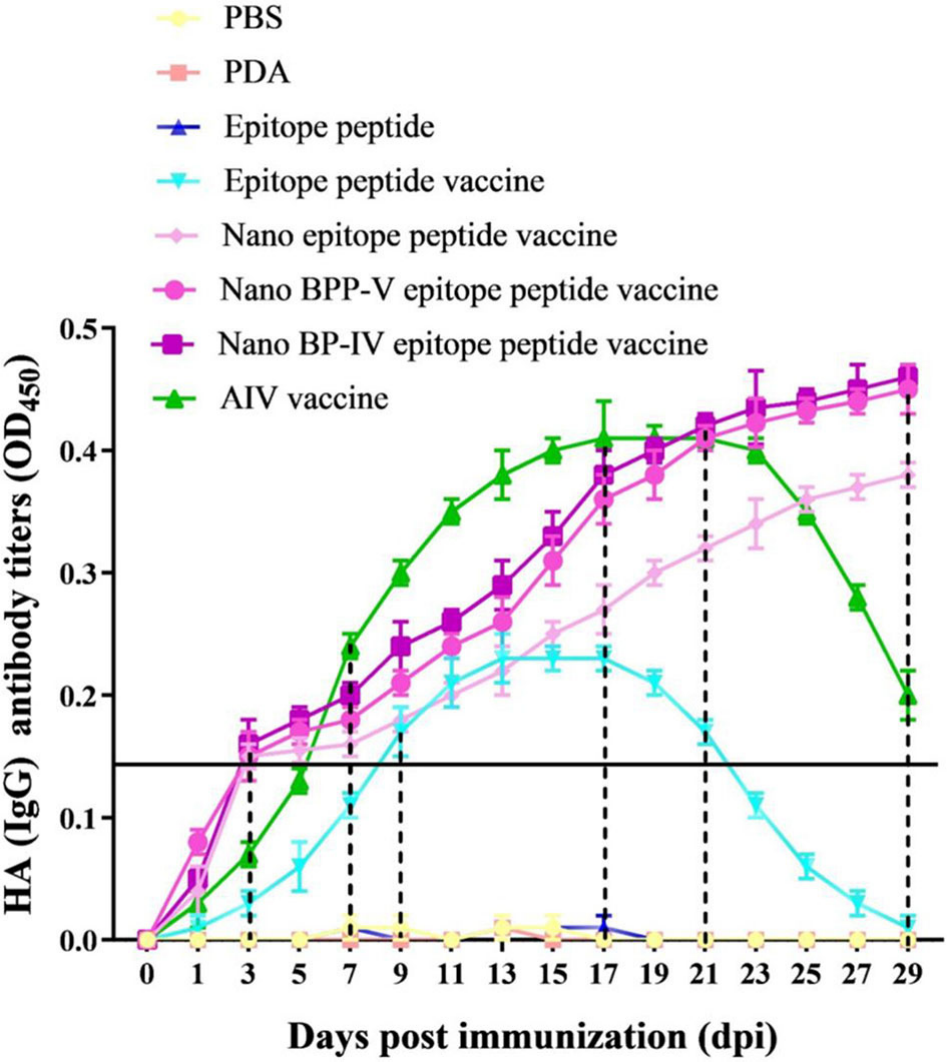
Changes in the anti-HA (IgG) antibody levels in mouse serum after immunization. The mice were immunized on day 0, and the anti-HA (IgG) levels in immunized mouse serum were detected on days 1, 3, 5, 7, 9, 11, 13, 15, 17, 19, 21, 23, 25, 27, and 29 after the immunization to evaluate the antibody duration.

To evaluate the effects of the nano BPP-V and BP-IV epitope peptide vaccines on specific antibody production, the serum levels of anti-HA (IgG) antibody in immunized mice were detected continuously from day 1 to day 29. The anti-HA antibody (IgG) was not detected in the PBS, PDA, or epitope peptide groups but was detected in the epitope peptide vaccine group on day 9 after immunization, with the level increasing steadily from days 9 to 17 after immunization and then decreasing continuously. In the commercial AIV vaccine group, the anti-HA (IgG) antibody was detected on day 7 after immunization, with the level increasing continuously from day 7 to day 21 after immunization and then obviously decreasing; however, the antibody was still detected on day 29 after immunization. Surprisingly, the antibody production in the nano epitope peptide vaccine and nano BPP-V and BP-IV epitope peptide vaccine groups was detected on day 3 after immunization, and the anti-HA antibody (IgG) level continued to increase from day 3 to day 29 after immunization (**Figure 4**). These results indicated that nano epitope peptide vaccines could promote the secretion of anti-HA antibody by using PDA as the carrier. Nano BPP-V and BP-IV epitope vaccines could rapidly stimulate the production of anti-HA antibody and prolong the immune duration.

### The Nano BPP-V and BP-IV Epitope Peptide Vaccines Can Promote the Secretion of Specific Antibodies (IgG, IgG1, IgG2a)

**Figure 5:**
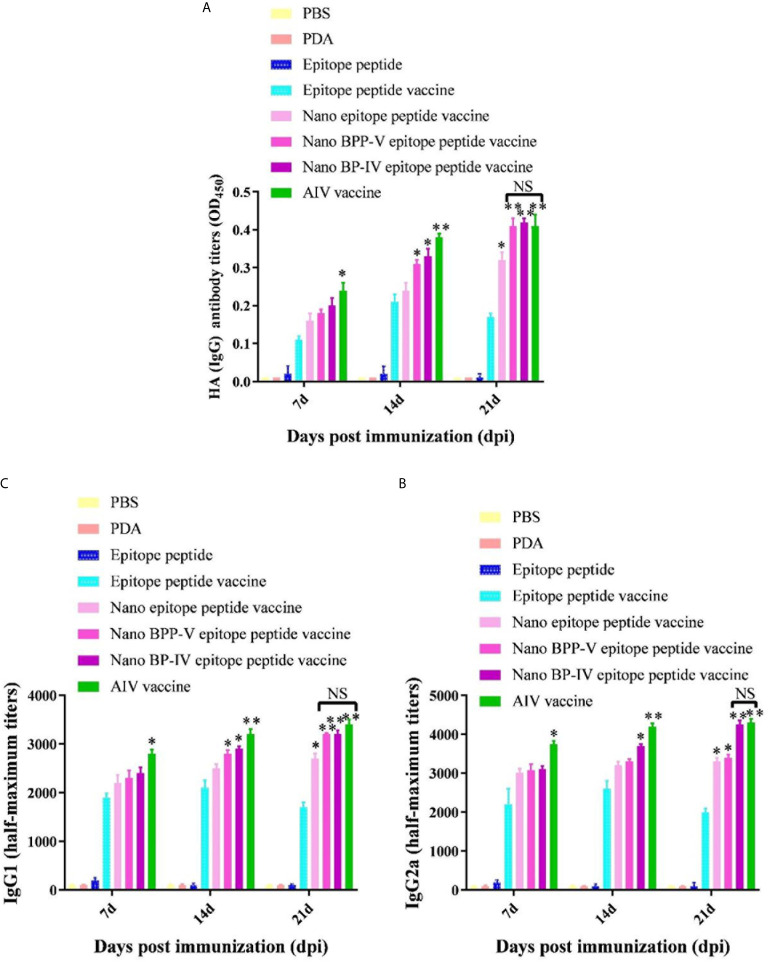
The nano BPP-V and BP-IV epitope peptide vaccines promoted the secretion of anti-HA (IgG) **(A)**, IgG1 **(B)** and IgG2a **(C)** antibodies in the sera of immunized mice. The immunized mouse serum samples were collected on days 7, 14, and 21 after immunization, and the levels of anti-HA (IgG), IgG1, and IgG2a were detected by ELISA method. *P < 0.05 and **P < 0.01, compared with the epitope peptide vaccine group. NS indicates no significant difference.

Mouse serum samples were randomly collected from each group (n = 5) to detect the levels of the anti-HA (IgG), IgG1 and IgG2a antibodies on days 7, 14 and 21 after the immunization. Neither PDA nor the epitope peptide group could effectively induce the production of anti-HA (IgG), IgG1 and IgG2a antibodies (**Figure 5**). The level of anti-HA (IgG) in the epitope peptide vaccine group was higher than that in the single epitope peptide group and increased from days 7 to 14 after immunization. The production of the anti-HA (IgG) antibody (*P* < 0.05) on day 7 after immunization was increased in only the AIV vaccine group compared with the epitope peptide vaccine group. Anti-HA (IgG) antibody secretion was significantly stimulated in the nano BPP-V and BP-IV epitope peptide vaccine (*P* < 0.05) and AIV vaccine (*P* < 0.01) groups on day 14 after immunization. The anti-HA (IgG) antibody level in the nano epitope peptide group was higher than that in the epitope peptide vaccine group (*P* < 0.05), and the anti-HA (IgG) antibody level in the nano BPP-V and BP-IV epitope vaccine and AIV vaccine groups were significantly higher than that in the epitope peptide vaccine group on day 21 after immunization (*P* < 0.01) (**Figure 5A**).

The two most abundant IgG subtypes (IgG1 and IgG2a) were also detected, and the IgG1 results are shown in **Figure 5B**. The IgG1 antibody production was significantly stimulated in the nano epitope peptide vaccine group compared with the epitope peptide vaccine group on day 21 after the immunization (*P* < 0.05). However, the levels of IgG1 in the nano BPP-V and BP-IV epitope peptide vaccine groups were higher than those in the epitope peptide vaccine group (14 days, *P* < 0.05; 21 days, *P* < 0.01), and were even similar to those in the AIV vaccine group. The IgG2a detection trends differed from those of IgG1 (**Figure 5C**). The production of the IgG2a antibody was significantly induced in the nano epitope peptide vaccine and nano BPP-V epitope peptide vaccine groups compared with the epitope peptide vaccine group on day 21 after immunization (*P* < 0.05). The IgG2a antibody level was significantly increased in the nano BP-IV epitope peptide vaccine group on day 14 after immunization (*P* < 0.05), and the vaccine significantly promoted the production of IgG2a on day 21 after immunization (*P* < 0.01). Notably, there was no significant difference in the IgG2a antibody level between the nano BP-IV epitope peptide vaccine group and the AIV vaccine group on day 21 after immunization.

These results indicated that the PDA modified nano BPP-V and BP-IV epitope peptide vaccines had a more obvious stimulation effect on the production of antigen-specific antibodies. The nano BPP-V and BP-IV epitope peptide vaccines can significantly induce the production of anti-HA(IgG), IgG1, and IgG2a antibodies, and the induction effect of Nano BP-IV epitope peptide vaccine was similar to those in the commercial AIV vaccine on day 21 after immunization.

### The Nano BPP-V and BP-IV Epitope Peptide Vaccines Can Induce Splenic Lymphocyte Proliferation

**Figure 6:**
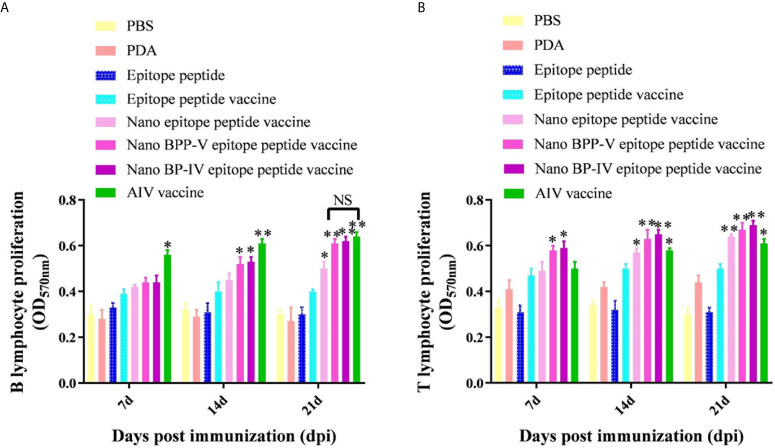
The nano BPP-V and BP-IV epitope peptide vaccines promoted the proliferation of splenic lymphocytes in immunized mice. The spleens of mice were collected on days 7, 14 and 21 after the first immunization (n = 5), and B lymphocyte **(A)** and T lymphocyte **(B)** proliferation activity were detected by the MTT method. The epitope peptide vaccine group was used as a control group, *P < 0.05; **P < 0.01. NS indicates no significant difference.

Mouse spleens (n = 5) were randomly collected from each group on days 7, 14 and 21 after immunization, and lymphocytes were then harvested to assess proliferation activity by the MTT assay. As shown in **Figure 6A**, B lymphocyte proliferation was not stimulated in the PDA and epitope peptide, while the B lymphocyte proliferation activity in the epitope peptide vaccine group was increased slightly. The proliferation of B lymphocyte was significantly increased in the nano epitope peptide vaccine group compared with the epitope peptide vaccine group on day 21 after immunization (*P* < 0.05). The nano BPP-V and BP-IV epitope peptide vaccines also significantly promoted the proliferation activity of B lymphocytes compared with the epitope peptide vaccine group (14 days, *P* < 0.05, 21 days, *P* < 0.01). The commercial AIV vaccine significantly induced the proliferation of B lymphocytes on day 7 after immunization (*P* < 0.05), and the activity was significantly increased on days 14 and 21 after immunization (*P* < 0.01). Remarkably, the B lymphocyte proliferation activities in the nano BPP-V and BP-IV epitope peptide vaccine groups were not significantly different from those in the AIV vaccine group on day 21 after immunization.

Interestingly, PDA stimulated the proliferation of T lymphocytes to some extent (**Figure 6B**). Only the nano BPP-V and BP-IV epitope peptide vaccines significantly promoted the proliferation of T lymphocytes compared with that in the epitope peptide vaccine group on day 7 after immunization (*P* < 0.05). The nano epitope peptide vaccine and AIV vaccine significantly induced the proliferation of T lymphocytes (*P* < 0.05), and the nano BPP-V and BP-IV epitope peptide vaccines significantly induced the proliferation of T lymphocytes on day 14 after immunization (*P* < 0.01). The proliferation of T lymphocytes was significantly stimulated by the AIV vaccine (*P* < 0.05), the nano epitope peptide vaccine and the nano BPP-V and BP-IV epitope peptide vaccines on day 21 after immunization (*P* < 0.01). These results indicated that the nano BPP-V and BP-IV epitope peptide vaccines could significantly promote the proliferation of splenic lymphocytes. The B lymphocyte proliferation activities in these groups did not differ from that in the AIV vaccine group on day 21 after immunization, while the T lymphocyte proliferation activities in the nano BPP-V and BP-IV epitope peptide vaccine groups were better than that in the AIV vaccine group on days 7, 14 and 21 after the immunization.

### The Nano BPP-V and BP-IV Epitope Peptide Vaccines Can Enhance Cellular Immune Response

**Figure 7:**
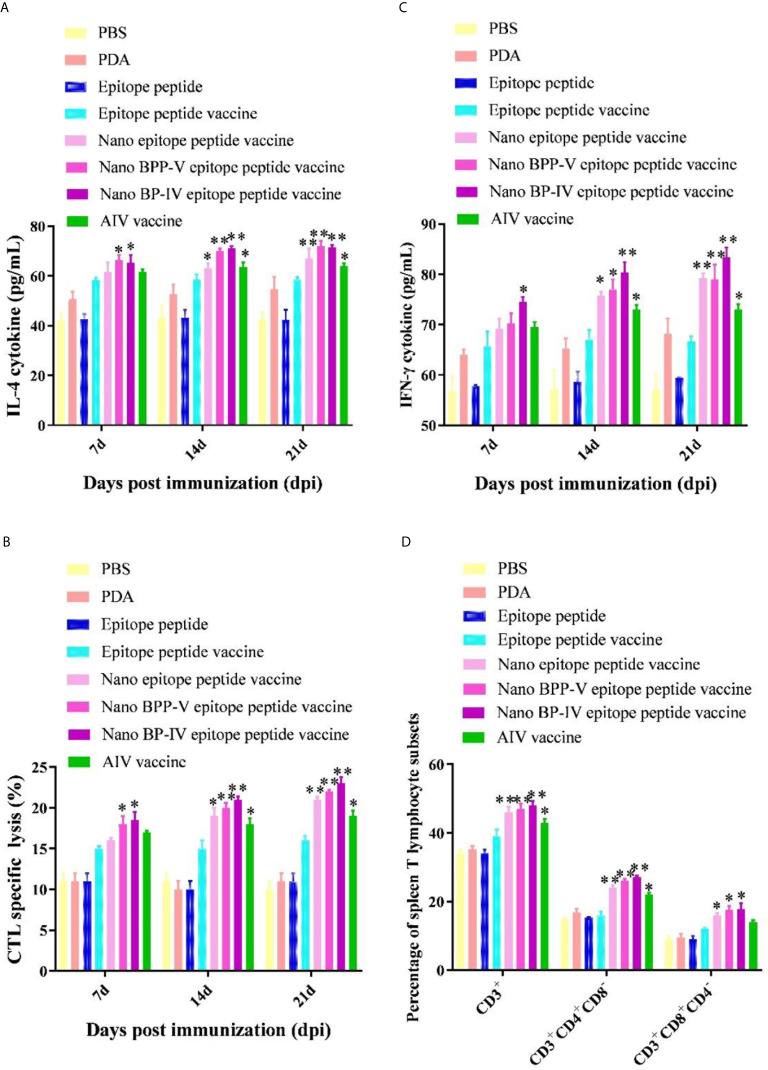
Effects of the nano BPP-V and BP-IV epitope vaccines on the cellular immune responses in immunized mice. The serum levels of IL-4 **(A)** and IFN-γ **(B)** in immunized mice were detected on days 7, 14, and 21 after immunization. The splenic CTL responses **(C)** were assessed on days 7, 14, and 21 after immunization, and splenic T lymphocyte typing **(D)** was performed on day 21 after the immunization. *P < 0.05, **P < 0.01, compared with the epitope peptide vaccine group.

The IL-4 and IFN-γ levels were detected to evaluate the immune effect of nano BPP-V and BP-IV epitope peptide vaccines, the results are shown in **Figures 7A, B**. The secretion of IL-4 and IFN-γ in the epitope peptide group was similar to that in the PBS group. Interestingly, the use of PDA alone increased IL-4 and IFN-γ secretion to a certain extent. Compared with the epitope peptide vaccine, the nano epitope peptide vaccine significantly induced the secretion of IL-4 (14 days, *P* < 0.05; 21 days, *P* < 0.01). The nano BPP-V and BP-IV epitope peptide vaccines significantly promoted the production of IL-4 on day 7 after immunization (*P* < 0.05) and significantly increased the level of IL-4 on days 14 and 21 after immunization (*P* < 0.01). The AIV vaccine group significantly promoted the production of IL-4 on days 14 and 21 after immunization (*P* < 0.05). In addition, only the nano BP-IV epitope peptide vaccine significantly promoted the level of IFN-γ compared with that in the epitope peptide vaccine group on day 7 after immunization (*P* < 0.05). However, the IFN-γ levels in the nano epitope peptide vaccine, nano BPP-V epitope peptide vaccine, nano BP-IV epitope peptide vaccine (*P* < 0.01) and AIV vaccine (*P* < 0.05) were significantly higher than that in the epitope peptide vaccine group on day 21 after immunization.

The CTL responses are shown in **Figure 7C**. Compared with the epitope peptide vaccine group, the nano epitope peptide vaccine significantly enhanced the CTL responses (14 days, *P* < 0.05; 21 days, *P* < 0.01). The CTL responses in the nano BPP-V and BP-IV epitope peptide vaccine groups were significantly enhanced compared to those in the epitope peptide vaccine group on day 7 after immunization (*P* < 0.05) and extremely significantly enhanced compared to those in the epitope peptide vaccine group on days 14 and 21 after immunization (*P* < 0.01). The CTL response was significantly enhanced by the AIV vaccine on days 14 and 21 after immunization (*P* < 0.05). In addition, compared with the epitope peptide vaccine, the nano epitope peptide vaccine and nano BPP-V and BP-IV epitope peptide vaccines not only enhanced the proportion of CD3^+^CD4^+^ T lymphocytes (*P* < 0.01) but also promoted the differentiation of CD3^+^CD8^+^ T lymphocytes (*P* < 0.05) on day 21 after immunization. The AIV vaccine group significantly enhanced the proportion of CD3^+^ and CD3^+^CD4^+^ T lymphocytes on day 21 after immunization (*P* < 0.05) (**Figure 7D**).

These results indicated that nano BPP-V and BP-IV epitope peptide vaccines could promote cellular immune response in immunized mice by enhancing cytokine secretion levels, CTL response, and T lymphocyte proportion.

### The Nano BPP-V and BP-IV Epitope Peptide Vaccines Can Alleviate Lung Damage in Immunized Mice

**Figure 8:**
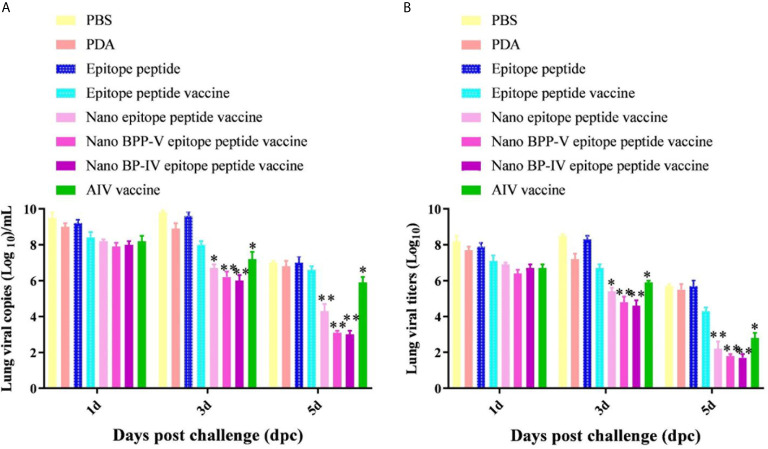
Detection of viral titres in the lungs of H9N2 AIV-challenged mice by RT-PCR and TCID_50_. Mice were challenged on day 21 after immunization, and their lungs were collected on days 1, 3 and 5 after the challenge. **(A, B)** show the genomic copy numbers and titres of H9N2 AIV in the mouse lungs on days 1, 3 and 5 post-challenge, respectively. The data are presented as the means ± SD. *P < 0.05 and **P < 0.01, compared with the epitope peptide vaccine group.

The lungs of immunized mice were harvested on days 1, 3 and 5 after the challenge, the pulmonary toxicity was detected on days 1, 3, and 5 after the challenge. The viral genome copy numbers and viral titres in the lungs of mice in the nano epitope peptide vaccine and AIV vaccine groups were significantly lower than those in the epitope peptide vaccine group on day 3 after the challenge (*P* < 0.05), and the pulmonary toxin loads in the nano BPP-V and BP-IV epitope vaccine groups were significantly lower than that in the epitope peptide vaccine group (*P* < 0.01). However, the nano epitope peptide vaccine, nano BPP-V epitope vaccine, and nano BP-IV epitope vaccine significantly decreased the viral genome copy numbers and viral titres in the lung tissues of immunized mice at day 5 after the challenge (*P* < 0.01), and viral genome copy numbers and viral titres in the AIV vaccine group were significantly lower than those in the epitope peptide vaccine group (*P* < 0.05). These results suggested that PDA, as a vaccine delivery carrier, could effectively clear the viral load of lung tissues (**Figure 8**).

**Figure 9:**
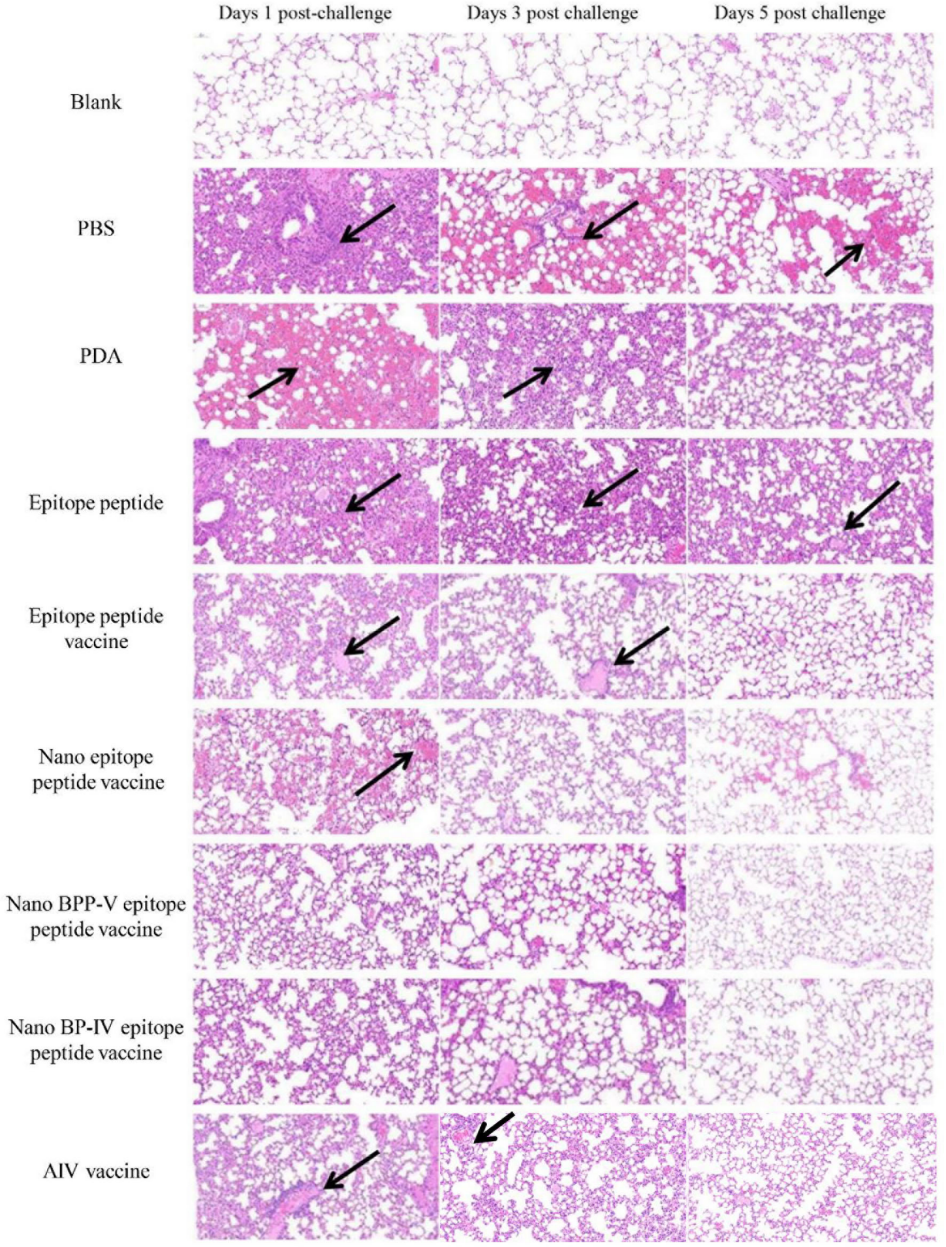
Representative histopathological images of processed lung sections on days 1, 3 and 5 after the challenge. Mice were challenged on day 21 after immunization, and their lungs were collected on days 1, 3 and 5 after the challenge. Lung sections were stained with H&E (n = 5). The arrows indicate lesion characteristics, including congestion, lymphocytic infiltration, microvascular embolism and hemorrhage.

To further clarify the protective effects of the nano BPP-V and BP-IV epitope peptide vaccines, mouse lung tissue sections of mice were harvested on days 1, 3 and 5 after the challenge for pathological observation, and lesion scores were recorded according to the degree of pathological injury. No pathological injuries were observed in the lung of mice in the blank group, while the lungs of mice in the other immunization groups showed different degrees of pathological injury. Over time, the pathological injuries were gradually reduced. On day 5 after the challenge, the lung tissues of mice in the PBS group were seriously damaged and showed massive hemorrhaging. A thickened alveolar wall and partially complete alveolar structure were observed in the lung tissues of mice in the PDA group. No clear alveolar structure, thickened alveolar walls and narrowed alveolar cavities were observed in the lung tissues of mice in the epitope peptide group. However, the pathological injuries in the epitope peptide vaccine, nano epitope peptide vaccine, and nano BPP-V and BP-IV epitope peptide vaccine groups were significantly alleviated. In particular, no bleeding and clearly visible alveolar structures were observed in the nano BPP-V and BP-IV epitope peptide vaccine groups. A small amount of inflammatory cell infiltration was observed in the AIV vaccine group (**Figure 9**).

**Figure 10:**
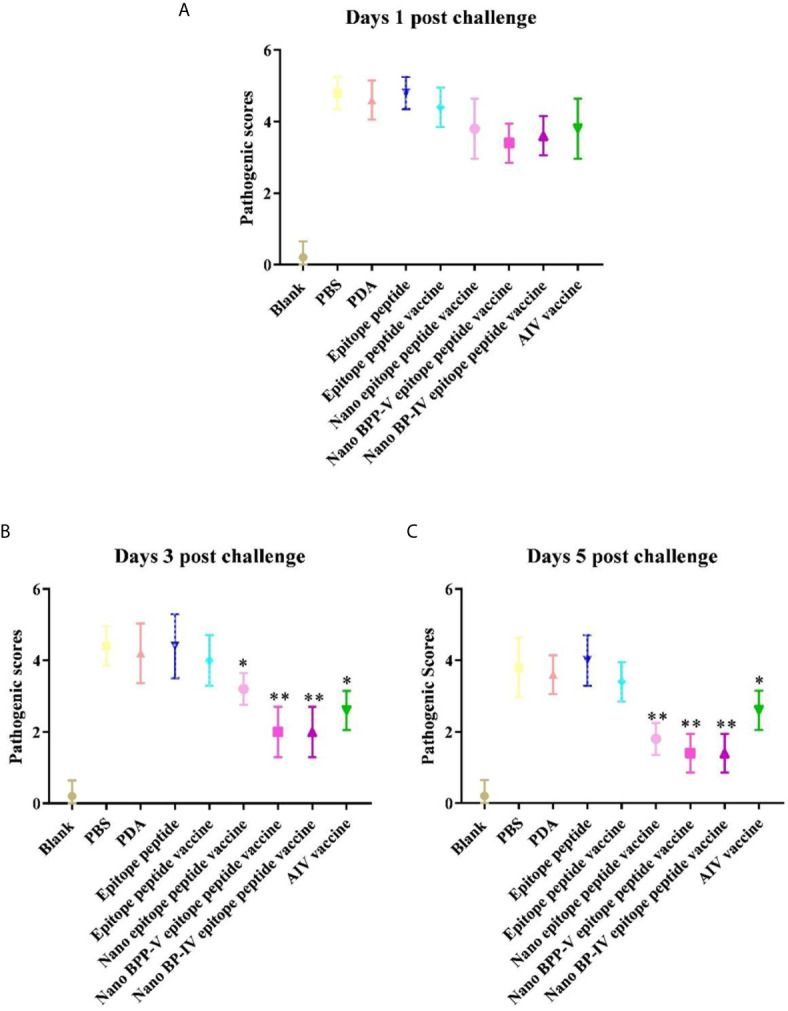
Pathological scores of processed lung sections on days 1, 3 and 5 after the challenge. The lungs of mice were collected from each group (n = 5) and sectioned to evaluate the pathological scores. The scoring standard was as follows: 0, no pathology; 1, local pulmonary congestion and inflammatory cell infiltration; 2, pulmonary congestion and inflammatory cell infiltration affecting 10% of the section; 3, pulmonary congestion and inflammatory cell infiltration affecting 20% of the section; 4, pulmonary congestion and inflammatory cell infiltration affecting 20-50% of the section; and 5, pulmonary congestion and inflammatory cell infiltration affecting > 50% of the section. *P < 0.05 and **P < 0.01, compared with the epitope peptide vaccine group. **(A)** Pathological scores of lung sections on day 1 after the challenge. **(B)** Pathological scores of lung sections on day 3 after the challenge. **(C)** Pathological scores of lung sections on day 5 after the challenge. Data represent the mean ± SD (n = 5).

The nano epitope vaccine, nano BPP-V epitope peptide vaccine and nano BP-IV epitope peptide vaccine effectively reduced the pulmonary viral load and pathological damage on day 5 after the challenge, and the pathological scores supported these results. There were no significant differences among all groups on day 1 after the challenge. Compared with those of mice in the epitope peptide vaccine group, the pathological scores of mice in the nano epitope peptide vaccine and AIV vaccine groups were significantly decreased (*P* < 0.05); the pathological scores of mice in the nano BPP-V and BP-IV epitope peptide vaccine groups were significantly decreased on day 3 after the challenge (*P* < 0.01). Notably, the pathological scores of mice in the nano epitope peptide vaccine, nano BPP-V and BP-IV epitope peptide vaccines (*P* < 0.01) and AIV vaccine (*P* < 0.05) groups were significantly lower than that in the epitope peptide vaccine group on day 5 after the challenge (**Figure 10**). The average lesion scores of processed lung sections were exhibited in **Table S1**. These results indicated that nano BPP-V and BP-IV epitope peptide vaccines had a better immune protective effect on day 5 after the challenge.

## Discussion

Due to the high rate of antigenic variation on the surface of influenza antigens, influenza vaccines are frequently mismatched and their effectiveness decreased (29, 30). A universal influenza vaccine, with comparable or better efficacy to seasonal influenza vaccines, could provide broad cross-protection against different influenza viruses and eliminate the need for annual updates of vaccine strains, thereby preventing a possible influenza pandemic (8). Studies have reported that the addition of nanoparticles to vaccine formulations can enhance antigen immunogenicity (31), promote antigen-targeted delivery (32), and regulate the rate of antigen release (33). Due to the beneficial features of nanoparticles, by combining nanoparticles with conserved antigen epitope, a broadly protective universal influenza vaccine will be developed (34).

Herein, PDA-modified nanoinfluenza vaccines were prepared in an effort to improve their effectiveness and immune protection abilities. A conserved amino sequence containing both T and B cell epitopes of the H9N2 AIV haemagglutinin (HA) protein (GNCVVQCQTERGGLN) was first selected as the antigen epitope by immunoinformatics analysis, and the epitope peptide was then conjugated to appropriate adjuvants to enhance the immune response by the transfer and controlled release of antigens (35). Our previous studies have shown that BPP-V and BP-IV can be used as immune adjuvants in combination with the commercial H9N2 AIV inactivated vaccine to enhance humoral and cellular immune responses (17, 18). Therefore, BPP-V and BP-IV were conjugated to synthesize the BPP-V and BP-IV epitope peptides. Nanoparticles were then utilized to enhance the immunogenicity of the H9N2 AIV epitope vaccines. The application of different nanoparticles represents a new trend in influenza vaccine development (31). In this study, we selected a new versatile biomaterial, PDA, which is widely applied in cancer therapeutics, antibiosis, inflammation prevention, tissue repair, etc. and has broad application prospects as a drug delivery carrier (13). In addition, due to the simple and mild synthesis requirement of PDA, the particle size and film thickness of PDA-modified nanoparticles are highly controllable by altering the key parameters such as the pH, temperature, and concentration of dopamine (36). Finally, the immunogenicities and immune protective effects of PDA-modified nanovaccines were determined *in vitro* and *in vivo*.

Inactivated vaccines protect against AIV primarily by inducing homosubtypic humoural immunity against the HA protein (37). In mice, the IgG antibody isotype contributes the most to influenza virus neutralization (38). Therefore, the serum levels of anti-HA (IgG) antibody in immunized mice were detected continuously. The antibody level in the nano epitope peptide vaccine and nano BPP-V and BP-IV epitope peptide vaccines could be detected on day 3 after immunization, and continued to increase from day 3 to day 29 after immunization. Additional experiments were needed to determine when the level peaked and how long antibody production lasted. However, antibody level in the epitope peptide vaccine and AIV vaccine groups was detected on day 9/7 after the immunization, respectively, and reached the peak on day 13/17 by single immunization. PDA-modified nanoparticles are known to have good biocompatibility and can enhance the anchoring efficiencies of antigens and adjuvants (39, 40), which is potentially why the nano BPP-V and BP-IV epitope peptide vaccines rapidly activated the antibody immune response. In addition, nanoparticles represent an efficient delivery system that can enhance the immune cell uptake and continuously release antigen (41). In this study, PDA nanoparticle, as the vaccine carrier, could rapidly produce the anti-HA antibodies and prolong the immune duration of nano BPP-V and BP-IV epitope peptide vaccines, functioning as antigen storage.

The IgG and its subtypes IgG1 and IgG2a antibodies were detected on days 7, 14 and 21 after immunization. While PDA itself did not induce an antibody response, the nano epitope peptide vaccine significantly stimulated the production of the IgG, IgG1 and IgG2a antibodies on day 21 after the immunization, indicating that in the absence of a molecular adjuvant, PDA could induce a strong specific antibody response as the epitope vaccine carrier, thereby enhancing the epitope peptide immunogenicity. These results are consistent with those of a previous study showing that nanoparticles as vaccine carriers enhanced the efficacy of influenza vaccines and improved immune properties (42). In addition, the antibody response was increased more obviously in the presence of the molecular adjuvants BPP-V and BP-IV, and the antibody levels of IgG and IgG1 were significantly increased on day 14 after immunization and comparable to those of the AIV vaccine group on day 21 after immunization. These results indicated that PDA, as a nanoparticle carrier, combined with molecular adjuvants of the bursa of Fabricius active peptide could synergistically enhance the immunogenicities of the nano BPP-V and BP-IV epitope peptide vaccines and induce strong humoural immune responses.

Studies have reported that CD4^+^ and CD8^+^ T cells play an important role in the immune response to viruses (43, 44). Therefore, cellular immune responses, such as IL-4, IFN-γ, CTL and T cell subtypes, were examined in this study. IL-4 drives the differentiation of Th2 cells, which support B cell activation and antibody production (45). IFN-γ can enhance the differentiation of T helper type 1 (Th1) cells, which activate the macrophages, natural killer (NK) cells, and cytotoxic T lymphocytes (CTLs) (46). The nano epitope peptide and nano BPP-V and BP-IV epitope peptide vaccines strongly promoted the production of IL-4 and IFN-γ, indicating that they induced the activation of Th1 and Th2 cells. Notably, the level of IL-4 in the nano BPP-V and BP-IV epitope peptide vaccine groups was higher than that in the AIV vaccine group on day 7 after immunization, and the IL-4 level continued to increase steadily over time. In addition, the nano BPP-V and BP-IV epitope peptide vaccines also enhanced the CTL response and increased the proportion of CD3^+^, CD4^+^ and CD8^+^ T cells in immunized mice. These results indicated that PDA nanoparticles, as vaccine carriers, conjugated to T and B cell epitope of the H9N2 AIV HA protein promoted IL-4 and IFN-γ secretion, enhanced the CTL response, and improved T lymphocyte differentiation, thereby controlling viral replication. These results were therefore consistent with those of previous studies (47, 48).

To evaluate the immuneprotective effects of the nano epitope vaccines, mice were challenged with H9N2 AIV three weeks after immunization. Because H9N2 AIV is a low-pathogenicity virus that commonly infects wild birds but does not cause obvious clinical symptoms or even death after challenge (49). Therefore, the pulmonary toxicity was detected on days 1, 3, and 5 after the challenge. The nano epitope vaccine and nano BPP-V and BP-IV epitope peptide vaccines could effectively reduce pulmonary viral load and pathological damage on day 5 after the challenge, which was also suggested by the pathological scores. These results suggest that the nano epitope peptide vaccine and the nano BPP-V and BP-IV epitope peptide vaccines can accelerate viral clearance from the lungs and reduce pathological damage, thereby providing effective protection against AIV. This may due to the antigen-targeted delivery of PDA. PDA nanoparticles can be used as carriers for drugs or imaging agents, and PDA-modified drug nanoparticles can effectively target tumor sites by enhancing the permeability and retention effects and reducing drug leakage during drug delivery (50, 51). Moreover, the immunoprotective effects of nano BPP-V and BP-IV epitopes peptide vaccines observed after challenge may be mediated through humoral and cell-mediated immune responses. Because compared to the epitope peptide vaccine, nano BPP-V and BP-IV epitopes peptide vaccines significantly increased anti-HA (IgG), IgG1 and IgG2a antibodies levels, cytokine level, CTL response and T lymphocyte differentiation on day 21 after the immunization. The viral titres was also significantly decreased on day 5 after the challenge, although it was not fully known, the lung viral titers may be correlated with anti-HA (IgG), IgG1 and IgG2a antibodies response. Because the HA antibody level in the nano BPP-V and BP-IV epitope peptide vaccines could be still detected on day 29 after the immunization. It has been reported that an adjuvant that induced mixed IgG1 and IgG2a responses may be effective at reducing RSV lung titers and reducing disease severity (52). In addition, the immuneprotective effects of the nano BPP-V and BP-IV epitope peptide vaccines were better than that of the commercial AIV vaccine in mice. However, these vaccines can elicit the secondary immune effects that are comparable to those of the commercial vaccine in poultry, or even reduce the mortality rate of AIV, as well as the underlying immune mechanism remain to be further studied.

## Conclusion

In conclusion, nano BPP-V and BP-IV epitope vaccines were successfully prepared by the coupling of the BPP-V and BP-IV epitope peptides with PDA. The nano BPP-V and BP-IV epitope vaccines, especially the nano BP-IV epitope vaccine, quickly induced antibody production and a sustained immune response, promoted humoural and cellular immune responses, reduced viral lung damage and provided effective protection against AIV virus infection. This study revealed that the use of PDA as a carrier can improve the immunogenicities and delivery efficiencies of influenza vaccines, thereby providing a theoretical basis for the design and development of PDA as a carrier of new nanoinfluenza vaccines.

## Data Availability Statement

The original contributions presented in the study are included in the article/**Supplementary Material**. Further inquiries can be directed to the corresponding authors.

## Ethics Statement

The animal study was reviewed and approved by Animal Experiment Committee of Henan University of Science and Technology.

## Author Contributions

CW and CL conceived the idea and guided the whole study. YL, JZ, TS, and LC carried out the experiments. JZ and SS provided comments on the experimental results. YL wrote the draft. YL and CW revised the manuscript. XW and MZ provided experiment guidance and resources. All authors contributed to the article and approved the submitted version.

## Funding

The whole study was supported from the financial support from the National Natural Science Foundation of China (grant number U2004151 and 31802159) and PhD Start-up Fund of Henan University of Science and Technology (13480071).

## Conflict of Interest

The authors declare that the research was conducted in the absence of any commercial or financial relationships that could be construed as a potential conflict of interest.

## Publisher’s Note

All claims expressed in this article are solely those of the authors and do not necessarily represent those of their affiliated organizations, or those of the publisher, the editors and the reviewers. Any product that may be evaluated in this article, or claim that may be made by its manufacturer, is not guaranteed or endorsed by the publisher.
