# Supplementary material for: Development of PDA Nanoparticles for H9N2 Avian Influenza BPP-V/BP-IV Epitope Peptide Vaccines: Immunogenicity and Delivery Efficiency Improvement
Source: Front Immunol. 2021 Jul 27;12:693972. doi: 10.3389/fimmu.2021.693972 (PMC8353371; doi:10.3389/fimmu.2021.693972)
Supplement: Supplementary file 1 [file Table_1.docx]

***Supplementary Materials***

**Supplementary Table 1** The average lesion scores of processed lung sections

| Scores Post challenge (d) | Blank | PBS | PDA | Epitope peptide | Epitope peptide vaccine | Nano epitope peptide vaccine | Nano BPP-V epitope peptide vaccine | Nano BP-IV epitope peptide vaccine | AIV vaccine |
| --- | --- | --- | --- | --- | --- | --- | --- | --- | --- |
| 1 | 0.2 | 4.8 | 4.6 | 4.8 | 4.4 | 3.8 | 3.4 | 3.6 | 3.8 |
| 3 | 0.2 | 4.4 | 4.2 | 4.4 | 4 | 3.2 | 2 | 2 | 2.6 |
| 5 | 0.2 | 3.8 | 3.6 | 4 | 3.4 | 1.8 | 1.4 | 1.4 | 2.6 |

Five mice in each group were scarificed for each time point, and the lungs were collected to stained with H&E, then scored according to the pathological damage. The data presents the average of the five scores in each group.
